# The Four Key Genes Participated in and Maintained Atrial Fibrillation Process *via* Reprogramming Lipid Metabolism in AF Patients

**DOI:** 10.3389/fgene.2022.821754

**Published:** 2022-05-20

**Authors:** Yijin Fang, Yue Wu, Liangming Liu, Huadong Wang

**Affiliations:** ^1^ Department of Pathophysiology, Key Laboratory of State Administration of Traditional Chinese Medicine of the People’s Republic of China, School of Medicine, Jinan University, Guangzhou, China; ^2^ State Key Laboratory of Trauma, Burns and Combined Injury, Shock and Transfusion Department of Research Institute of Surgery, Daping Hospital, Army Medical University, Chongqing, China

**Keywords:** atrial fibrillation, metabolomics bioinformatics, gene set enrichment analysis (GSEA), key gene, lipid metabolism

## Abstract

Atrial fibrillation (AF) is always in high incidence in the population, which can lead to serious complications. The structural and electrical remodeling of atrial muscle induced by inflammatory reaction or oxidative stress was considered as the major mechanism of AF. The treatment effect is not ideal based on current mechanisms. Recent studies demonstrated that lipid metabolism disorder of atrial muscle played an important role in the occurrence of AF. What key genes are involved is unclear. The purpose of the present study was to explore the lipid metabolism mechanism of AF. With the GEO database and the genomics of AF patients, metabolic related pathways and the key genes were analyzed. At the same time, the rat model of cecal ligation and puncture (CLP) was used to confirm the results. GSE 31821 and GSE 41177 were used as data sources, and the merged differentially expressed genes (DEGs) analysis showed that a total of 272 DEGs were found. GO annotation, KEGG, and gene set enrichment analysis (GSEA) showed that the fatty acid metabolism and the lipid biosynthetic process were involved in AF. Cholesterol biosynthesis, arachidonic acid metabolism, and the lipid droplet pathway were obviously increased in AF. Further analysis showed that four key genes, including ITGB1, HSP90AA1, CCND1, and HSPA8 participated in pathogenesis of AF regulating lipid biosynthesis. In CLP rats, metabolic profiling in the heart showed that the pyrimidine metabolism, the biosynthesis of unsaturated fatty acid metabolism, arginine and proline metabolism, and the fatty acid biosynthesis were involved. The four key genes were confirmed increased in the heart of CLP rats (*p* < 0.05 or 0.01). The results suggest that the lipid metabolism disorder participates in the occurrence of AF. ITGB1, HSP90AA1, CCND1, and HSPA8 are the key genes involved in the regulation of lipid biosynthesis.

## Introduction

Atrial fibrillation (AF) is the most common arrhythmia, and there are about 33.5 million patients suffering from atrial fibrillation worldwide (Lloyd-Jones et al., 2004). In Europe, there are 8.8 million adult patients suffering from AF over the age of 55 (Krijthe et al., 2013), and there are nearly 2.3 million patients in the United States (Benjamin et al., 2009). The incidence of AF is about 1–2% in the general population in China. The prevalence of AF was 0.71% in China among the general population in 2018, and it was 2.35% in elder people over 75 years old (Wang et al., 2018). Persistent AF may lead to serious complications, of which the most harmful is systemic ischemic stroke with its high mortality and disability rate. In addition, AF can also lead to heart failure, myocardial infarction, renal dysfunction, Alzheimer’s disease, and cognitive decline (Huang et al., 2018). The new guidelines issued by 2020ACC/AHA/EACTS put forward a comprehensive ABC approach for the diagnosis and treatment of AF: A—anticoagulation/avoid stroke, B—better symptom management and better control of heart rate and rhythm through drugs and other measures, and C—optimization of cardiovascular complications and strengthening the management of other complications and lifestyle (Yoon et al., 2019; Hindricks et al., 2020). These approaches played a certain role in the prevention and treatment of complications and risk factor management of AF. However, the critical problems involving in the bleeding risk of antithrombotic therapy, the aggravation of arrhythmia by antiarrhythmic drugs, the complications of ablation therapy, and the high recurrence rate are not resolved. Intensive study for the mechanism of AF is needed to acquire the targeted measures.

Several mechanisms of AF were put forward in the past studies. It was considered that the structural and electrical remodeling of atrial muscle was the basis for the occurrence and maintenance of AF (Nguygen et al., 2013), in which the inflammatory response and abnormal gene expression were the triggering factors. Previous studies found that some proteins or genes such as SPP1 and FCGR3B were involved in the process of AF. Moreover, recent studies found that the disorder of myocardial energy metabolism played a vital role in the process of AF. Myocardial energy metabolism alteration played an important role in the atrial structural and electrical remodeling of AF (Kim et al., 2013). 70% of the energy support of the heart comes from the fatty acid β oxidation in the condition of physiology; the energy provided by glucose metabolism only accounts for 10∼30%, and the rest comes from the metabolism of lactic acid, ketone body, and amino acid. AF presented critical enzymes of glucose oxidation upregulation in a short time. Chronic atrial fibrillation presented more energy metabolism disturbances (Lenski et al., 2015). It is unclear what genes participate in the regulation of myocardial energy metabolism and the occurrence and development of AF.

**FIGURE 1:**
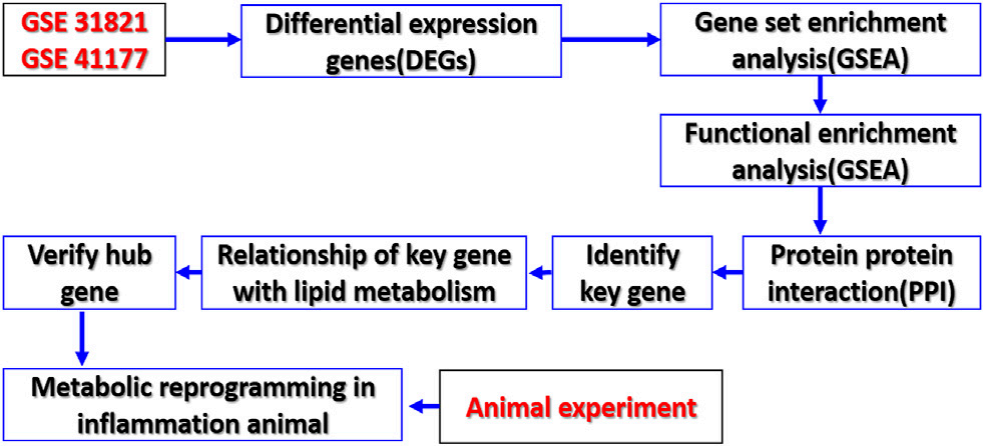
Study flowchart. Sequencing data from AF patients and controls in GSE 31821 and GSE 41177 datasets were analyzed by bioinformatics to identify metabolic reprogramming and its key genes of AF.

With the gene data of patients with AF through the GEO database, the key genes involved in lipid metabolism of AF and the relationship of these genes to the metabolic changes of AF were analyzed. At the same time, the CLP rat model as the trigger factor of AF was used to detect the changes of myocardial metabolomics and related genes (Figure 1). The study will provide a new direction for the prevention and treatment of AF.

## Materials and Methods

### Animal Ethics

Animal experiments were performed following the principles of the Guide for the Care and Use of Laboratory Animals set forth by the United States National Institutes of Health (NIH Publications, 8th edition, 2011). The protocol was approved by the Research Council and Animal Care and Use Committee of the Research Institute of Surgery, Army Medical University (2021-7-16).

### Inflammation Model Establishment in Rats

Sprague-Dawley (SD) rats (female and male) weighing 200–220 g were obtained from the Experimental Animal Center of the Research Institute of Surgery, Daping Hospital, Army Medical University. Animals were bred with a 12:12 h dark/light cycle under filtered positive-pressure ventilation. A temperature of 23–25°C and a relative humidity of 55–65% were maintained in the breeding room, respectively. The rats were fasted except for water *ad libitum* for 8 h prior to the initiation of surgery.

The sodium pentobarbital (45 mg/kg, i. p.) was used for anesthetizing rats for the surgical procedure. The cecal ligation and puncture (CLP) was applied to reproduce systemic inflammatory response as described previously (Zhu et al., 2021). Briefly, the cecum was exposed carefully and ligated and punctured 0.7 cm from a distance with a triangular needle 1.5 mm in diameter, and fecal matter was allowed to flow into the abdominal cavity freely. Then the abdomen was closed, and the rats were put back in the cages with *ad libtitum* access to food and water. Myocardial tissues were harvested at 12 h after CLP and the samples were stored at −80°C until analysis.

### Data Acquisition and Differential Gene Analysis

In order to obtain the data of gene difference of cardiac tissue in AF patients, datasets in the GEO (Gene Expression Omnibus) database (https://www.ncbi.nlm.nih.gov/geo/) were obtained, GSE 31821 and GSE 41177 (Yeh et al., 2013), and the sample platforms used were GPL570 (HG-U133_Plus_2) Affymetrix Human Genome U133 Plus 2.0 Array. Linear models for the microarray data (LIMMA) software package were used for background correction and data normalization. On this foundation, the differentially expressed genes (DEGs) were analyzed using R package. The threshold was FC = 1.5, *p* < 0.05. It was considered that FC > 1.5, *p* < 0.05 was upregulated and FC < −0.5, *p* < 0.05 was downregulated. Ggplot2, a plotting system for R, was used to insert the package to make the volcano map.

### DEG Functional Enrichment Analysis

Gene set enrichment analysis (GSEA 4.1.0) was used to analyze the gene function. The molecular signatures database (MSigDB) of C2 (c2. cp. kegg. v7.4. symbols. gmt) was used as the reference gene sets in GSEA. The number of permutations was set to 1,000, and the phenotypic labels were high or low expression. FDR <0.25 and *p* < 0.05 were defined as the cut-off criteria to confirm significant gene sets. Enrichment results were obtained by Metascape online analysis (https://metascape.org/org/gp/index.html). The cellular components, molecular functions, biology, and involved signal pathways of DEGs were analyzed using DAVID online database, including GO enrichment [biological process (BP), cellular component (CC), molecular function (MF)] and Kyoto Encyclopedia of Genes and Genomes (KEGG); *p* < 0.05 was considered significant.

### Analysis of Key Gene

In order to obtain the key genes involved in the occurrence of AF, PPI (protein–protein interaction) analysis was carried out for the above obtained DEGs. The String database was used to analyze the interaction between proteins (https://string-db.org/) (Huang et al., 2019). Cytoscape 3.7.2 was further used (https://js.cytoscape.org) to analyze the key genes (Shannon et al., 2003). With Cytoscape 3.7.2, the centriscape APP plug-in was used to screen hub genes with degree value ≥ mean + SD as a node; betweeness value ≥ mean + SD was considered as a node to screen out bottleneck genes (Liu H. et al., 2020). Further MCODE APP plug-in was used to select core genes. According to parameters degree cutoff ≥ 3 and k-core ≥ 4, two clusters with the highest scores were selected and obtained the corresponding genes, namely, core genes. The bottleneck, hub, and core genes were further analyzed by the Venn graph to obtain key genes.

### Metabolics Profiling

Metabolomics analysis was performed in heart tissues of the inflammation model and the sham control by using an UHPLC system (Vanquish, Thermo Fisher Scientific) with a UPLC BEH Amide column (2.1 mm × 100 mm, 1.7 μm) coupled to Q Exactive HFX mass spectrometer (Orbitrap MS, Thermo). The QE HFX mass spectrometer was used for its ability to acquire MS/MS spectra on the information-dependent acquisition (IDA) mode in the control of the acquisition software (Xcalibur, Thermo). The resulting three-dimensional data involving the peak number, sample name, and normalized peak area were fed to the SIMCA14 software package (Umetrics, Umea, Sweden) for principal component analysis (PCA). The annotated metabolites were illustrated as a volcano plot. A chord diagram was applied for biomarker metabolites depicting distributions and links between potential metabolites. The expression levels of the significantly changed metabolites and metabolic pathway analysis between septic rats and the sham control were analyzed using heatmaps generated from TBtools and a bubble plot, respectively.

### Real-Time Quantitative RT-PCR

To further verify the key genes related lipid metabolism in AF, the RNA was extracted from rat myocardial tissues suffering from CLP using TRIzol reagent according to the manufacturer’s instructions (Cat#15596018, Thermo). cDNA was synthesized using a RT reagent Kit with gDNA Eraser (Perfect Real Time) for real-time quantitative qRT-PCR (Cat#RR047A, Takara). SYBR Premix Ex Taq II (TliRNaseH Plus) (Cat#RR820B, Takara) was applied to analyze mRNA expression of key genes. The relative RNA expression levels were calculated with the efficiency corrected 2^−ΔΔCT^ method using β-actin as an internal control. Gene specific primer pairs used in this experiment are listed in Table 1.

**TABLE 1:** PCR primer sequences of four key genes.

Genes	Forward primers	Reverse primers
ITGB1	CAT​CCC​AGC​AAG​TCC​CAA​GT	TCA​CAG​TGT​CTC​CCA​ACA​CG
HSPA8	GCA​CCC​AGG​CCA​GTA​TTG​AG	ACG​GAA​CAG​GTC​AGC​ATT​CA
CCND1	GCA​CCC​AGG​CCA​GTA​TTG​AG	GGG​TGG​GTT​GGA​AAT​GAA​C
HSP90AA1	GGC​AGC​AAA​GAA​ACA​CCT​GG	CTG​AAG​CCG​GAA​GAC​AGG​AG

### Statistical Analysis

All statistical analyses were performed using SPSS 17.0 (IBM, United States). The DEGs were identified using the Limma package with *p* < 0.05 and |Log(FC)|>0.58 as the cut-off criteria. Hierarchical clustering analysis was used for the identified featured genes by the heatmap package in R. The centriscape method was used to select the key genes. All statistical analyses were performed using R software (version 4.0.5, http://www.r-project.org). *p* < 0.05 was considered significant.

## Results

### Differentially Expressed Genes in Atrial Muscle From AF Patients

**FIGURE 2:**
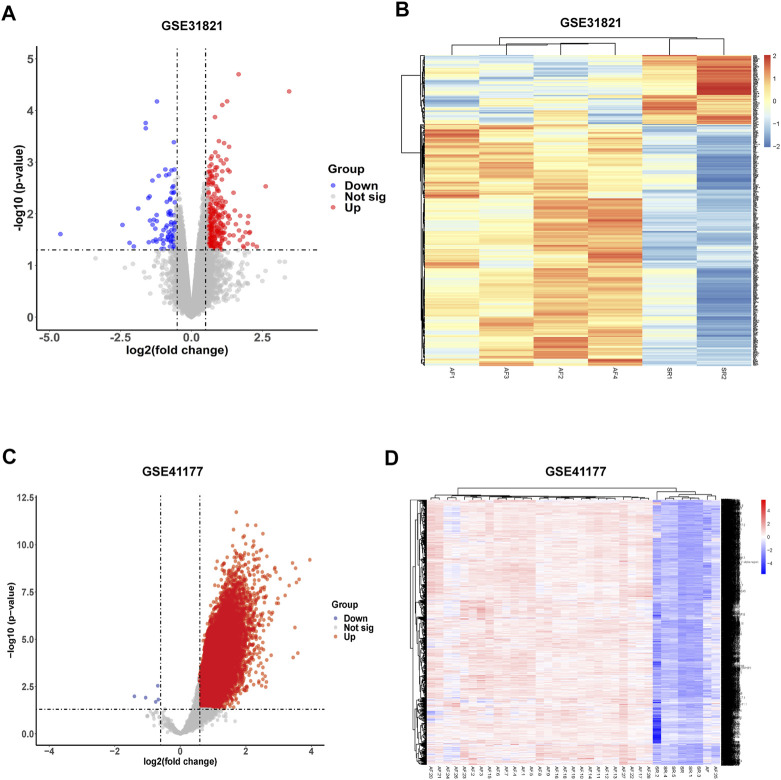
Identification of differentially expressed genes (DEGs) from GSE31821 and GSE 41177 datasets. **(A)** Volcano plot in GSE 31821. **(B)** Heatmap in GSE 31821. **(C)** Volcano plot in GSE 41177. **(D)** Heatmap in GSE 41177. Blue dots indicate downregulated DEGs while red dots indicate upregulated DEGs. Statistically significant DEGs were identified as those with a Student’s *t*-test *p*-value <0.05 and |log2(fold-change)|>0.585.

To acquire differentially expressed genes (DEGs) in AF, gene expression in atrial samples between healthy and AF patients were analyzed according to the GEO database. Three healthy and 3 AF atrial samples in GSE 31821 and 3 healthy and 16 AF in GSE 41177 were obtained. PCA analysis showed that the gene expression patterns were significantly different in atrial muscle between healthy people and AF patients both in GSE 31821 and GSE 41177. Compared with healthy samples, a total of 272 DEGs were found in merged analysis of GSE31821 and GSE 41177 (Figures 2A–D). Cluster analysis showed that there were significant differences between healthy atrial muscle and AF-derived atrial tissues.

### Functional Enrichment Analysis of DEGs

**FIGURE 3:**
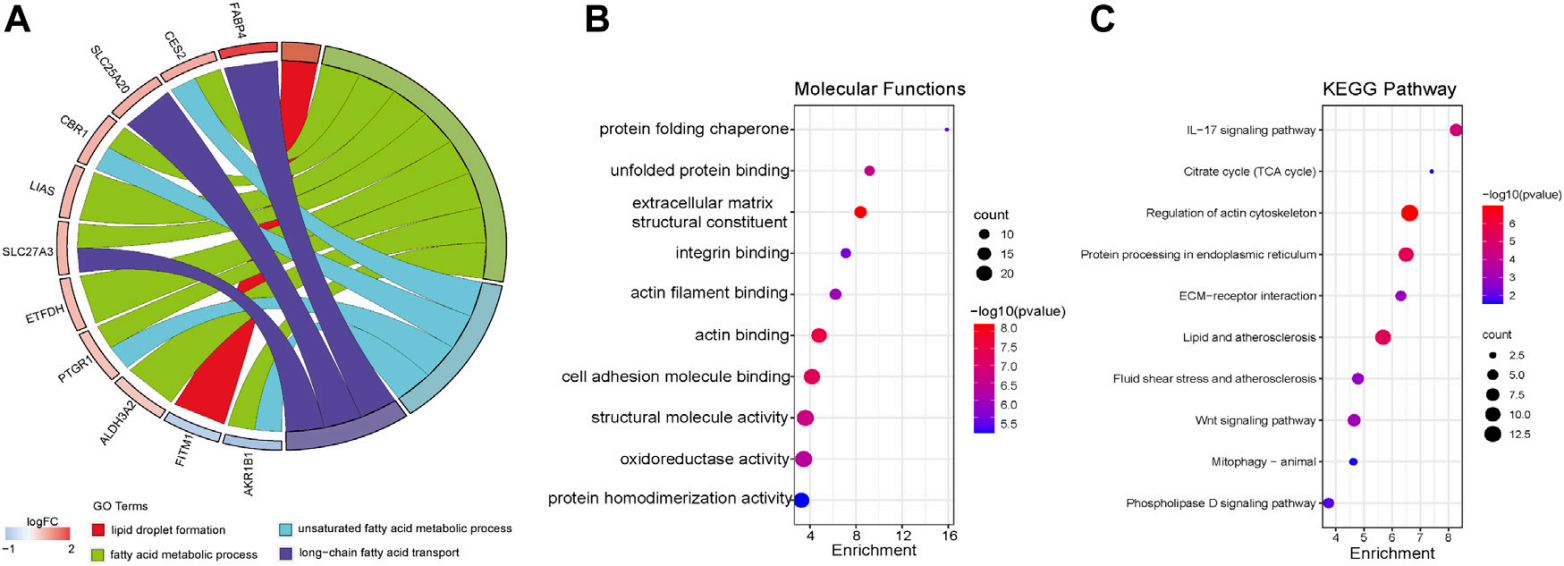
Functional enrichment analysis of DEGs using Metascape. **(A)** Biological process (BP). **(B)** Molecular function (MF). **(C)** Kyoto Encyclopedia of Genes Genomes (KEGG).

**FIGURE 4:**
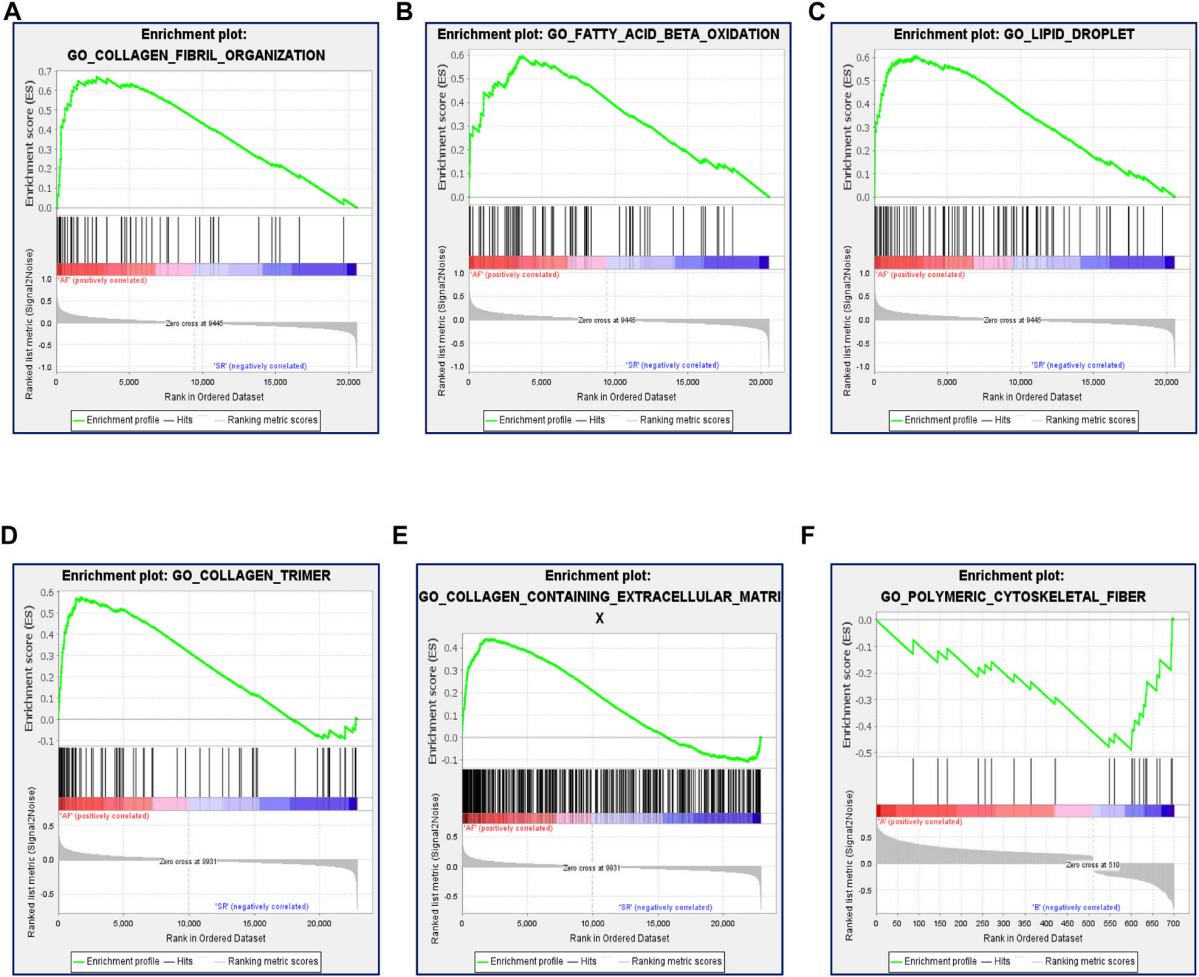
Function enrichment analysis of DEGs by GSEA. **(A)** Enrichment plot: GO_COLLAGEN_FIBRIL_ORGANIZATION. **(B)** Enrichment plot: GO_FATTY_ACID_BETA_OXIDATION. **(C)** Enrichment plot: GO_LIPID_DROPLET. **(D)** Enrichment plot: GO_COLLAGEN_TRIMER. **(E)** Enrichment plot: GO_ COLLAGEN_CONTAINING_EXTRACELLULAR_MATRIX. **(F)** Enrichment plot: GO_POLYMERIC_CYTOSKELETAL_FIBER.

Previous studies showed that many factors were involved in the occurrence and maintenance of AF, including the myocardial structure and electrical remodeling. Myocardial structural remodeling mainly included the increase of collagen caused by the structural change of myocardial fibroblasts. Recent studies found that the metabolic changes of heart tissue, especially the changes of fatty acid metabolism, played an important role in the occurrence and maintenance of AF. To explore the underlying functions of these DEGs in AF, enrichment analyses were performed using the DAVID 6.8 enrichment analysis and R package. GO annotation analysis showed these DEGs were involved in several processes, including angiogenesis and fibrosis. At the same time, the fatty acid metabolism and lipid biosynthetic process were involved. KEGG analysis was adopted to define the possible pathways linking the functions of these DEGs. Besides, actin and tight junction regulation pathways were involved, and several lipid-related metabolic pathways were remarkably enriched, including lipid droplet formation, unsaturated fatty acid metabolic process, and so on (Figure 3). Gene set enrichment analysis (GSEA) was then performed to further validate the pathways that were differentially enriched among DEGs. The significantly enriched signaling pathways were presented based on their normalized enrichment score. As expected, fatty acid ß-oxidation and the lipid droplet pathway were closely related besides AF-related collagen fibril organization, collagen trimer, and the collagen-containing extracellular matrix (Figures 4A–F). The results suggest that lipid metabolism disorder may play an important role in the occurrence of AF.

### Key Genes Participating in Lipid Metabolism Following AF

**FIGURE 5:**
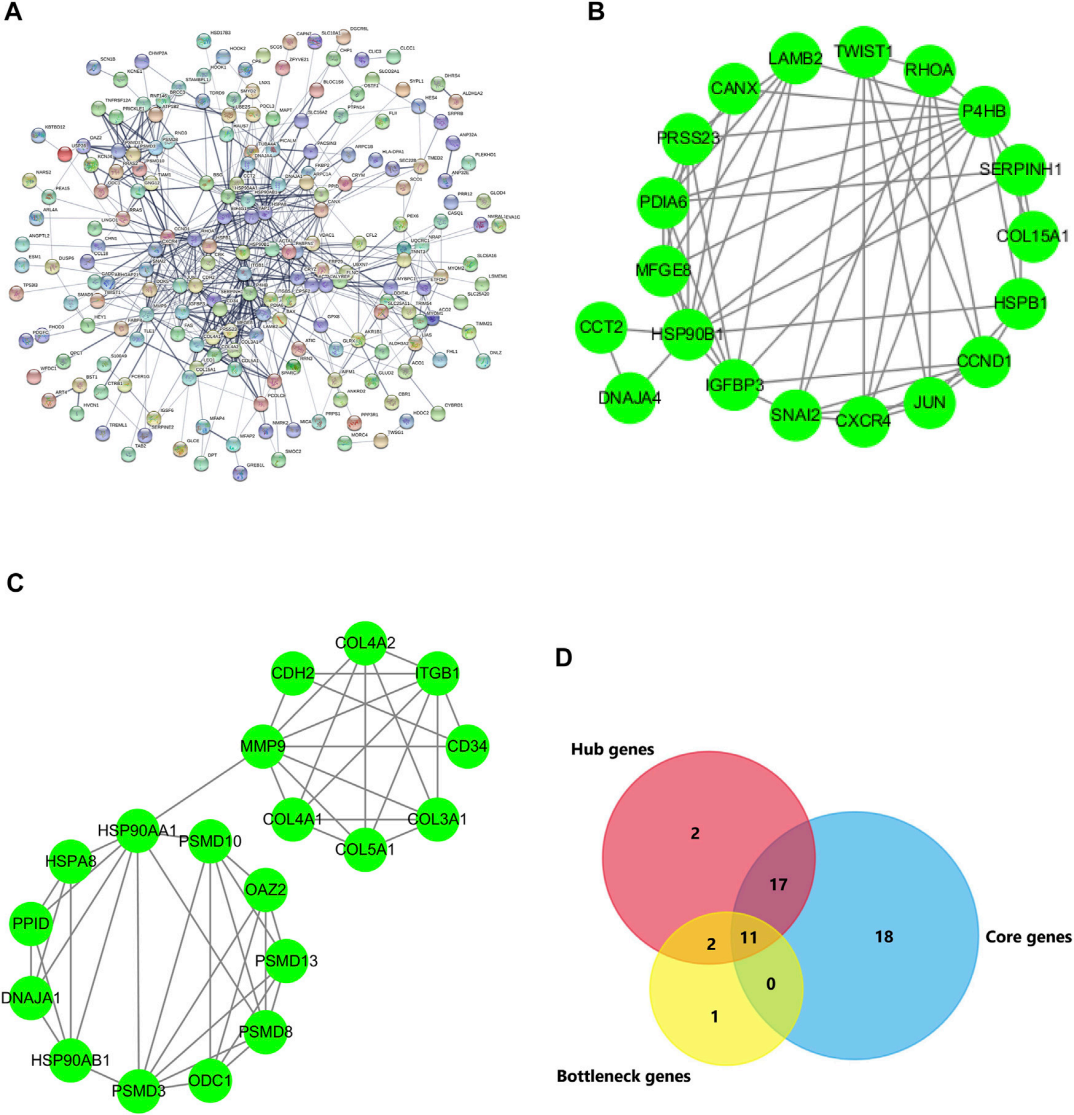
PPI network of DEGs and key genes. **(A)** DEGs with 201 nodes and 590 edges displayed using STRING 11.0. **(B,C)** The PPI network data from STRING was further analyzed using Cytoscape. The 32 hub genes and 14 bottleneck genes were screened using Cytoscape software plug-in Centiscape and 46 core genes were screened using MCODE plug-in. **(D)** The key genes are obtained by the intersection of hub genes, bottleneck genes, and core genes using Venn.

To look for key genes which take part in the modulating lipid metabolism in the occurrence of AF, a PPI network analysis was conducted using the STRING database. Based on Cytoscape3.7.2, the centiscape APP was used to analyze the hub genes (Degree Value ≥ Mean + SD). The results showed that there were 30 hub genes, including HSP90AA1, HSPA8, RHOA, MMP9, CCND1, P4HB, ITGB1, JUN, HSP90B1, HSPB1, CDH2, ACTA1, SNAI2, ACTN2, COL3A1, CXCR4, CANX, PSMD8, SERPINH1, COL4A2, HSP90AB1, CCT2, CD34, LAMB2, PDIA6, PSMD3, COL4A1, VDAC1, ARPC1A, PSMD10, SPARC, and IGFBP3 (Table 2; Figure 5A). At the same time, the genes were considered as bottleneck genes when betweenness value ≥ mean + SD. There were 12 bottleneck genes, including HSP90AA1, RHOA, MMP9, HSPA8, JUN, CCND1, ITGB1, ACTA1, P4HB, VDAC1, BSG, ACTN2, HSPB1, and CANX (Table 2). Core genes were investigated using Mcode APP (Degree Cutoff ≥3, K-core ≥4), and there were two highest clusters including 35 core genes, including MFGE8, PDIA6, PRSS23, HSP90B1, P4HB, CANX, SERPINH1, COL15A1, RHOA, SNAI2, HSPB1, CCND1, JUN, DNAJA4, CXCR4, TWIST1, LAMB2, CCT2, IGFBP3, PPID, COL3A1, HSP90AB1, HSP90AA1, HSPA8, PSMD8, PSMD10, PSMD3, PSMD13, COL4A2, COL4A1, MMP9, ODC1, OAZ2, DNAJA1, COL5A1, MYOM1, CDH2, TNNT3, MYBPC1, ACTA1, MYOM2, ITGB1, CFL2, NRAP, and ARPC1A CASQ1 (Table 2; Figures 5B,C).

**TABLE 2:** List of bottleneck genes, hub genes, and core genes.

	Genes
Bottleneck gene	HSP90AA1, RHOA, MMP9, HSPA8, JUN, CCND1, ITGB1, ACTA1, P4HB, VDAC1, BSG, ACTN2, HSPB1, CANX
Hub gene	HSP90AA1, HSPA8, RHOA, MMP9, CCND1, P4HB, ITGB1, JUN, HSP90B1, HSPB1, CDH2, ACTA1, SNAI2, ACTN2, COL3A1, CXCR4, CANX, PSMD8, SERPINH1, COL4A2, HSP90AB1, CCT2, CD34, LAMB2, PDIA6, PSMD3, COL4A1, VDAC1, ARPC1A, PSMD10, SPARC, IGFBP3
Core gene	MFGE8, PDIA6, PRSS23, HSP90B1, P4HB, CANX, SERPINH1, COL15A1, RHOA, SNAI2, HSPB1, CCND1, JUN, DNAJA4, CXCR4, TWIST1, LAMB2, CCT2, IGFBP3, PPID, COL3A1, HSP90AB1, HSP90AA1, HSPA8, PSMD8, PSMD10, PSMD3, PSMD13, COL4A2, COL4A1, MMP9, ODC1, OAZ2, DNAJA1, COL5A1, MYOM1, CDH2, TNNT3, MYBPC1, ACTA1, MYOM2, ITGB1, CFL2, NRAP, ARPC1A, CASQ1

**FIGURE 6:**
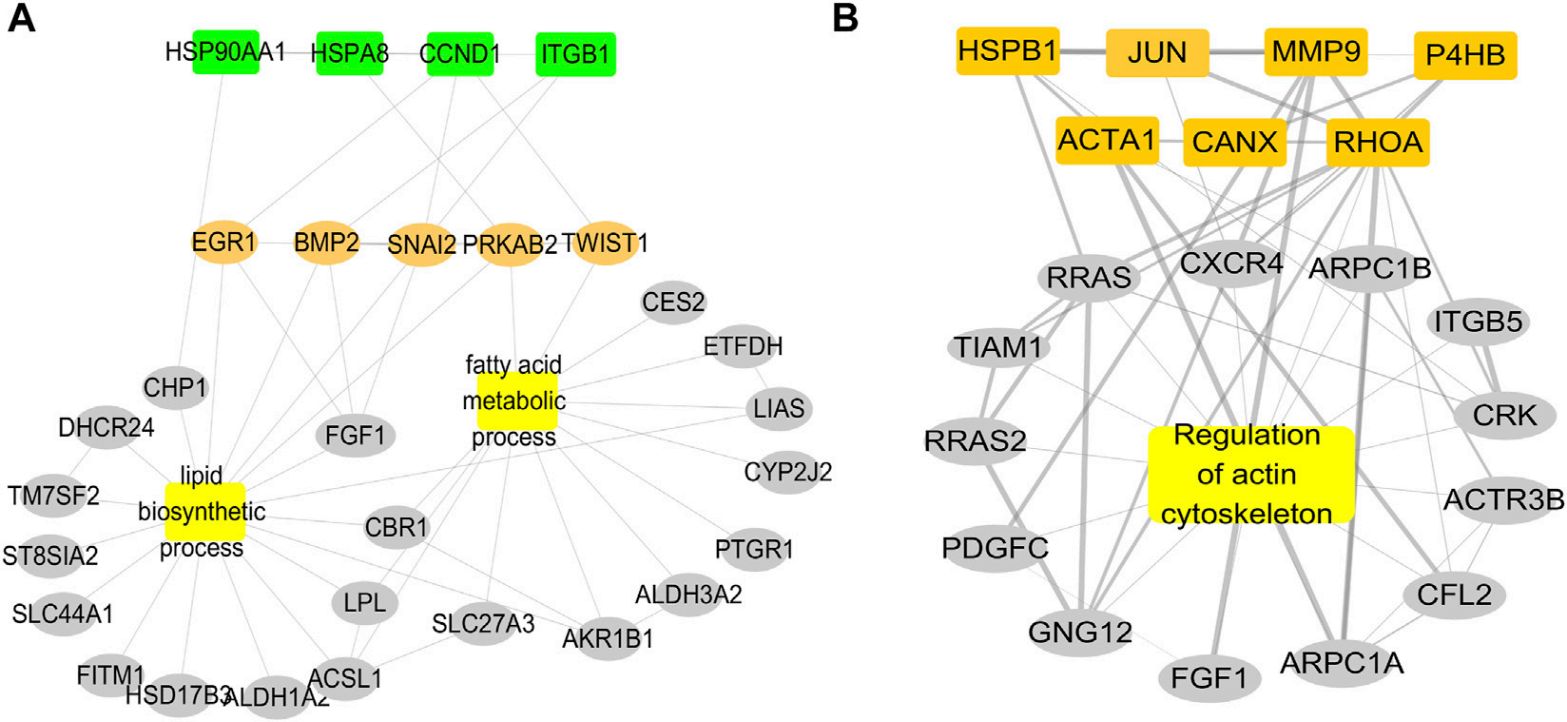
Relationship of key genes with the lipid biosynthetic process and the fatty acid metabolic process. **(A)** PPI network of four key genes enriched in fatty acid metabolism and fatty acid synthesis interact. **(B)** PPI network of other seven key genes enriched in the actin process.

To further search for the key genes, the hub genes, bottleneck genes, and core genes were made to intersect by the Venn analysis. 11 key genes were found, including P4HB, CANX, RHOA, HSPB1, CCND1, JUN, HSP90AA1, HSPA8, MMP9, ACTA1, and ITGB1 (Figure 5D). It is unclear which of these key genes participate in the lipid metabolism process following AF. The relationship of these key genes with lipid metabolism was further observed. The results showed that four key genes including CCND1, HSP90AA1, HSPA8, and ITGB1 were found interlinked with lipid metabolism and biosynthesis. Seven other genes majorly participated in the regulation of the actin process. Further analysis showed that four key genes participated in the fatty acid metabolic process and lipid biosynthetic process *via* TWIST1, SNAI2, PRKAB2, EGR1, and BMP2. The results suggest that there are four key genes including TWIST1, SNAI2, PRKAB2, EGR1, and BMP2 participating in the occurrence of AF *via* lipid metabolism (Figures 6A,B).

### Validation of Key Genes and Related Genes Expression

**FIGURE 7:**
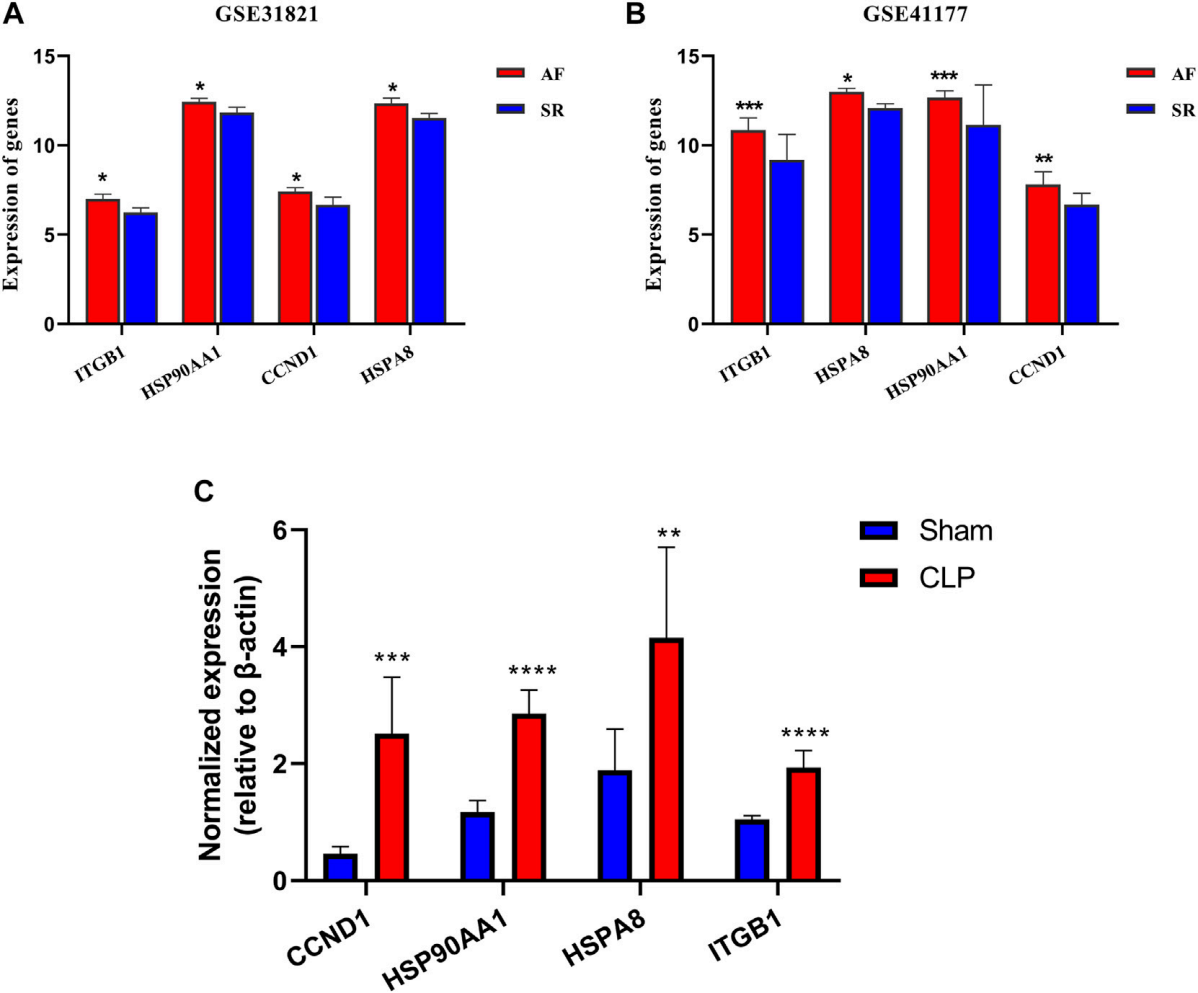
Verification of key genes in GSE31821 and GSE 41177 and CLP. **(A,B)** Validation of expression of genes and key genes in patients with AF and SR groups in GSE31821 and GSE41177. **(C)** The expressions of four key genes in a heart suffering from inflammatory rats *p < 0.05, **p < 0.01, ***p < 0.001, ****p < 0.0001.

To validate the role of key genes in AF, the expressions of key genes were observed, and the results showed that expression of the key genes ITGB1, HSP90AA1, CCND1, and HSPA8 was significantly increased in AF (*p* < 0.05) (Figures 7A,B). The results suggested that these key genes participated in the regulation of lipid metabolism in AF.

### The Metabolomics Profiling and Key Genes Expression in Hearts in Inflammation Rats

**FIGURE 8:**
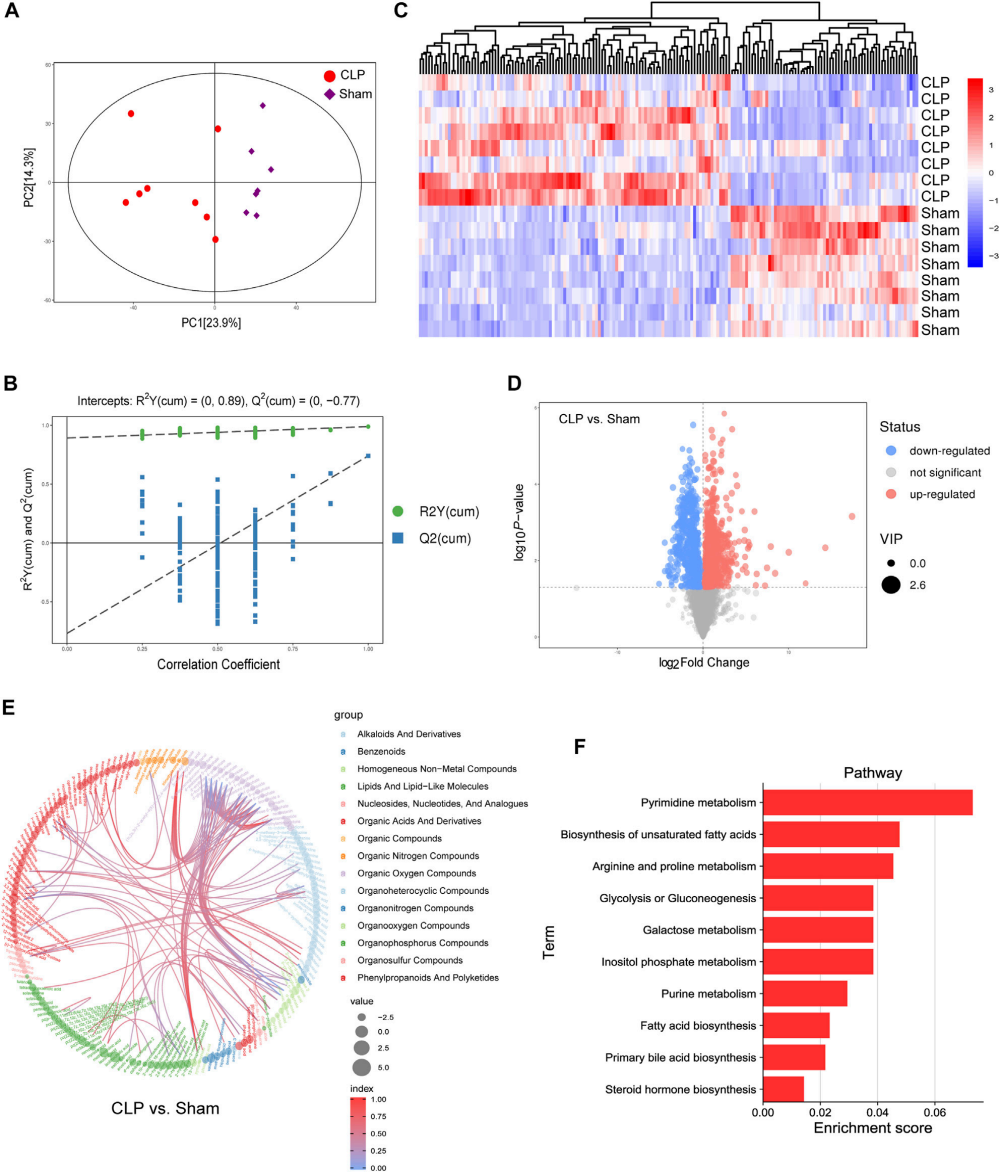
Landscape of the metabolism profile of the heart following in CLP. **(A)** Principal component analysis (PCA) score plot for metabolomics analysis in CLP and Sham. Orange represented CLP , and blue represent the Sham. **(B)** Permutation test of the OPLS-DA model for CLP group vs. Sham group. **(C)** Heat map analyzed using TBtools showing the significantly changed metabolites in CLP rats and the Sham control. **(D)** Volcano plot. **(E,F)** Chord and histogram and metabolic pathway enrichment analysis identified in CLP rats and the Sham control. The different color depths of circles represent the *p* value of pathway enrichment analysis.

To verify the metabolomics change following the AF, the untargeted metabolomics analysis was performed in heart tissue of CLP rats by LC-MS. The PCA score plot and volcano plot showed metabolite difference from discrimination analysis (Figures 8A–C). To compare the expression levels of significantly enriched metabolites between CLP rats and the sham control, an interactive heat map with TBtools was constructed. 173 metabolites were found to be significantly different (Figure 8D). The main different metabolites included lipids and lipid-like molecules, alkaloids and derivatives, homogeneous non-metal compounds, and so on. The main pathways were pyrimidine metabolism, biosynthesis of unsaturated fatty acid metabolism, arginine and proline metabolism, and fatty acid biosynthesis (Figures 8E,F).

To further confirm the expression of these key genes, their mRNA expression levels in myocardial tissues were determined between septic (*n* = 6) and healthy rats (*n* = 6) with quantitative real-time PCR. The results showed that the mRNA expression levels of the four genes in CLP rats were significantly higher than those in control rats (Figure 7C). The results validated the lipid metabolism disorders in the heart during inflammation, and there were four key genes involved.

## Discussion

As a common arrhythmia, AF has a high incidence rate and often induces many severe complications such as stroke. Previous studies demonstrated that atrial structural and electrical remodeling played a vital role in the occurrence and maintenance of AF. The present study found that metabolic reprogramming dominated by lipid metabolic disorder played an important role in the occurrence of AF. Eleven intersection genes were identified. Four lipid metabolism–related genes, including ITGB1, HSP90AA1, CCND1, and HSPA8, are found to be the key genes that participate in the occurrence and development of AF through metabolic reprogramming *via* regulating Twist1, Snai2, PRKAB2, EGR1, and BMP2. This study put forward the new mechanisms of AF, which provides a new direction for the prevention and treatment of AF.

In recent years, it was demonstrated that energy metabolism was significantly disturbed in the pathogenesis of AF. 70% of the energy comes from fatty acid β oxidation for a normal heart; the energy provided by glucose metabolism only accounts for 10∼30%, and the rest comes from the metabolism of lactic acid, ketone body, and amino acid (Sun et al., 2014). Previous studies showed that the glucose oxidation enzymes in cardiac myocytes were upregulated suffering from AF in a short time, and the energy metabolism of atrial myocytes was dysregulated in chronic atrial fibrillation (Wiersma et al., 2017). Previous studies found that the mechanism was related to the decrease in adenine nucleotides and the decrease in enzyme activity related to energy metabolism. Other studies (Shirihai et al., 2015) demonstrated that even transient AF rapidly reduced atrial contractility and diminished myocardial energy consumption. When AF was persistent, energy metabolism disorder could result in Ca^2+^ level overload (Dorn and Maack, 2013). Shirihai et al. (Wu et al., 2013) found that myocardial energy metabolism disorder could activate the activation of redox signals, which led to myocardial necrosis and cardiomyopathy and induced tissue inflammatory response and cardiomyocyte apoptosis, hypertrophy, and fibroblast activation and then accelerated myocardial remodeling, which might be an important mechanism for the progress of myocardial structural remodeling caused by AF. There was no definite mechanism for abnormal cardiac energy metabolism following AF. The present study found that many genes were significantly changed with abnormal lipid metabolism in the process of AF; among them, four key genes including ITGB1, HSP90AA1, CCND1, and HSPA8 participated in the occurrence of AF by regulating the process of lipid metabolism.

Previous studies confirmed that atrial structural and electrical remodeling were the theoretical basis for AF occurrence and maintenance. The atrial structural remodeling was mainly manifested in cell volume, glycogen accumulation, connexin expression, myolysis, and sarcoplasmic reticulum rupture. The changes of ion channels in atrial myocytes, such as sodium channel, potassium channel, and calcium channel, resulted in the disturbance of electrophysiological characteristics of atrial myocytes, which was the basis of electrical remodeling of AF (Kornej et al., 2015). Previous studies demonstrated that several factors could induce structural and electrical remodeling of atrial muscle, such as inflammation, oxidative stress, and so on. The infiltration of inflammatory cells and inflammatory factors released in myocardial tissue participated in the occurrence and development of AF (Zhang et al., 2013). Except for tumor necrosis factor-α, interleukin-6, and hypersensitive C-reactive protein, studies showed that galectin 3, matrix metalloproteinase 9, lipoprotein 2, and N-type pro-peptide type III collagen were also involved in the process of AF (Girmatsion et al., 2009). Oxidative stress and inflammatory response jointly accelerated myocardial remodeling.

According to the marked reprogramming of lipid metabolism in the myocardium of patients with AF, whether the inflammatory reaction as an important trigger factor of AF can result in a similar metabolic reprogramming, the systemic inflammatory response was reproduced by CLP and the changes of metabolic level of myocardial tissue were observed. The results showed that the metabolic reprogramming of myocardial tissue after the inflammatory response manifested the disorder of lipid-related metabolism; the increase in fatty acid synthesis was similar to the metabolism of AF. Four lipid metabolism–related genes were involved in the metabolic reprogramming. The results suggested that the metabolism reprogramming mediated by the four key genes may be the key mechanism of AF occurrence and persistence. In recent years, according to the importance of metabolic change in the occurrence and development of diseases, many studies confirmed that metabolic change was not only the initial response of the body to external stimuli but also an important intermediate bridge in the follow-up process of disease development (Karakikes et al., 2013). Therefore, metabolic change may provide a new target for early treatment and prevention of AF.

In addition to inflammation response and oxidative stress, previous studies showed that several micro RNAs were involved in the process of AF. For example, Barana A et al. found that the expression of miRNA-1 in myocardial tissue of patients with AF was significantly decreased, which induced AF occurrence through combinedly targeting the channel IK1 and connexin-43 (Barana et al., 2014). In addition, miRNA-1 may regulate the expression level of Ca^2+^ channel protein, reduces the release of Ca^2+^ from the sarcoplasmic reticulum, and triggers the electrical remodeling in patients with AF (Yamac et al., 2016). In addition to miRNA-1, the increase in expression of miRNA-21 in cardiomyocytes of patients with chronic AF (Yamac et al., 2016) led to the decrease in a voltage-dependent calcium channel subunit protein expression. The decreased expression of CACNB2 led to the decrease in L-type current, promoted myocardial fibrosis, and accelerated myocardial electrical remodeling. The other microRNA, such as miRNA-199a, was found to be related to AF. Our present study found that four key genes were involved in AF. To observe the relationship of four key genes with microRNA modulating lipid metabolism, further analysis was performed in the present study. The results showed that many microRNAs were involved in the processes of the four key genes regulating lipid metabolism. For example, miR-520-5p and miR-5003-3p were involved in the regulation of HSP90AA1/PRKAE. miR652-3P, miR-3974, miR-1264, and miR-3157 were involved in the regulation of ITGB1/GPAMin lipid metabolism (data not shown). But detailed mechanisms need to be further verified.

There were two other studies to investigate the key genes in the pathogenesis of AF as previously shown (Liu Y. et al., 2020; Liu et al., 2021). Although we all used the same two datasets, GSE 31821 and GSE 41177, since the analysis methods and other datasets we used were not the same, we obtained different results. In the first study, the datasets GSE 41177, GSE 79768, and GSE 14975 were used to analyze the target genes *via* gene ontology and pathway enrichment. The results showed that the DEGs were primarily related to the plasma membrane and exosomes (Liu Y. et al., 2020), and the enriched DEGs were mostly in the receptor activity, MHC class I receptor activity, and MHC class II receptor activity. They identified six key genes including FCGR3B, CLEC10A, FPR2, IGSF6, S100A9, and S100A12 *via* PPI, which were majorly related to immune response and cell communication. In the second study, the datasets GSE79768, GSE31821, GSE115574, GSE14975, and GSE41177 were selected (Liu et al., 2021). With the PPI network, they identified SPP1, COL5A1, and VCAN as key genes which were associated with the extracellular matrix. According to the new mechanisms participating in the occurrence of AF, lipid metabolism disorder was a vital mechanism resulting in AF. So our present study was to explore the key genes related to lipid metabolism in the occurrence of AF. We found four key genes including ITGB1, HSP90AA1, CCND1, and HSPA8 playing an important role in the AF *via* regulating lipid metabolism. Our findings provided a new idea to find the key genes and new targets for the treatment of AF.

In conclusion, the lipid metabolic reprogramming of atrial muscle plays an important role in the occurrence and development of AF, and the increase in lipid metabolism is the main feature. ITGB1, HSP90AA1, CCND1, and HSPA8 are the key genes that participate in the occurrence and development of AF through regulating the metabolic reprogramming.

## Data Availability

The datasets presented in this study can be found in online repositories. The names of the repository/repositories and accession number(s) can be found below: https://www.ncbi.nlm.nih.gov/, GSE31821; https://www.ncbi.nlm.nih.gov/, GSE41177.

## References

[B1] Barana A. Matamoros M. Dolz-Gaitón P. Pérez-Hernández M. Amorós I. Núñez M. (2014). Chronic Atrial Fibrillation Increases MicroRNA-21 in Human Atrial Myocytes Decreasing L-Type Calcium Current. Circ. Arrhythmia Electrophysiol. 7 (5), 861–868. 10.1161/circep.114.001709 25107449

[B2] Benjamin E. J. Chen P.-S. Bild D. E. Mascette A. M. Albert C. M. Alonso A. (2009). Prevention of Atrial Fibrillation. Circulation 119 (4), 606–618. 10.1161/circulationaha.108.825380 19188521 PMC2635942

[B3] Dorn G. W. Maack C. (2013). SR and Mitochondria: Calcium Cross-Talk between Kissing Cousins. J. Mol. Cell Cardiol. 55, 42–49. 10.1016/j.yjmcc.2012.07.015 22902320

[B4] Girmatsion Z. Biliczki P. Bonauer A. Wimmer-Greinecker G. Scherer M. Moritz A. (2009). Changes in microRNA-1 Expression and Ik1 Up-Regulation in Human Atrial Fibrillation. Heart Rhythm 6, 1802–1809. 10.1016/j.hrthm.2009.08.035 19959133

[B5] Hindricks G. Potpara T. Dagres N. Arbelo E. Bax J. J. Blomström-Lundqvist C. (2020). 2020 ESC Guidelines for the Diagnosis and Management of Atrial Fibrillation Developed in Collaboration with the European Association of Cardio-Thoracic Surgery (EACTS)(EACTS). Eur. Heart J. 42 (5), 373–498. 10.1093/eurheartj/ehaa612 32860505

[B6] Huang C. X. Zhang S. Hua W. Huang D. J. (2018). Current Knowledge and Management Recommendations of Atrial Fibrillation: 2018. Chin J. Card. Pacing Electrophysi 32 (4), 315–367. 10.13333/j.cnki.cjcpe.2018.04.001

[B7] Huang G.-Z. Wu Q.-Q. Zheng Z.-N. Shao T.-R. Lv X.-Z. (2019). Identification of Candidate Biomarkers and Analysis of Prognostic Values in Oral Squamous Cell Carcinoma. Front. Oncol. 9, 1054. 10.3389/fonc.2019.01054 31681590 PMC6813197

[B8] Karakikes I. Chaanine A. H. Kang S. Mukete B. N. Jeong D. Zhang S. (2013). Therapeutic Cardiac-Targeted Delivery of miR-1 Reverses Pressure Overload-Induced Cardiac Hypertrophy and Attenuates Pathological Remodeling. J. Am. Heart Assoc. 2 (2), e000078. 10.1161/JAHA.113.000078 23612897 PMC3647279

[B9] Kim S. M. Kim J. M. Shin D. G. Kim J. R. Cho K. H. (2013). Relation of Atrial Fibrillation (AF) and Change of Lipoproteins: Male Patients with AF Exhibited Severe Pro-Inflammatory and Pro-Atherogenic Properties in Lipoproteins. Clin. Biochem. 47 (10-11), 869–875. 10.1016/j.clinbiochem.2013.10.026 24201066

[B10] Kornej J. Schmidl J. Ueberham L. John S. Daneschnejad S. Dinov B. (2015). Galectin-3 in Patients with Atrial Fibrillation Undergoing Radiofrequency Catheter Ablation. PLoS One 10 (4), e0123574. 10.1371/journal.pone.0123574 25875595 PMC4398460

[B11] Krijthe B. P. Kunst A. Benjamin E. J. Lip G. Y. H. Franco O. H. Hofman A. (2013). Projections on the Number of Individuals with Atrial Fibrillation in the European Union, from 2000 to 2060, from 2000 to 2060. Eur. Heart J. 34 (35), 2746–2751. 10.1093/eurheartj/eht280 23900699 PMC3858024

[B12] Lenski M. Schleider G. Kohlhaas M. Adrian L. Adam O. Tian Q. (2015). Arrhythmia Causes Lipid Accumulation and Reduced Glucose Uptake. Basic Res. Cardiol. 110 (4), 40. 10.1007/s00395-015-0497-2 26018791

[B13] Liu H. Zhou Q. Wei W. Qi B. Zeng F. Bao N. (2020). The Potential Drug for Treatment in Pancreatic Adenocarcinoma: A Bioinformatical Study Based on Distinct Drug Databases. Chin. Med. 15, 26. 10.1186/s13020-020-00309-x 32206083 PMC7079489

[B14] Liu L. Yu Y. Hu L. L. Dong Q. B. Hu F. Zhu L. J. (2021). Potential Target Genes in the Development of Atrial Fibrillation: A Comprehensive Bioinformatics Analysis. Med. Sci. Monit. 27, e928366. 10.12659/msm.928366 33741890 PMC7989062

[B15] Liu Y. Tang R. Zhao Y. Jiang X. Wang Y. Gu T. (2020). Identification of Key Genes in Atrial Fibrillation Using Bioinformatics Analysis. BMC Cardiovasc. Disord. 20 (1), 363. 10.1186/s12872-020-01653-4 32778054 PMC7419195

[B16] Lloyd-Jones D. M. Wang T. J. Leip E. P. Larson M. G. Levy D. Vasan R. S. (2004). Lifetime Risk for Development of Atrial Fibrillation. Circulation 110 (9), 1042–1046. 10.1161/01.cir.0000140263.20897.42 15313941

[B17] Nguygen T. N. Hilmer S. N. Cumming R. G. (2013). Review of Epidemiology and Management of Atrial Fibrillation in Developing Countries. Int. J. Cardiol. 167 (6), 2412–2420. 10.1016/j.ijcard.2013.01.184 23453870

[B18] Shannon P. Markiel A. Ozier O. Baliga N. S. Wang J. T. Ramage D. (2003). Cytoscape: A Software Environment for Integrated Models of Biomolecular Interaction Networks. Genome Res. 13 (11), 2498–2504. 10.1101/gr.1239303 14597658 PMC403769

[B19] Shirihai O. S. Song M. Dorn G. W. (2015). How Mitochondrial Dynamism Orchestrates Mitophagy. Circ. Res. 116 (11), 1835–1849. 10.1161/circresaha.116.306374 25999423 PMC4443843

[B20] Sun D.-M. Yuan X. Wei H. Zhu S.-J. Zhang P. Zhang S.-J. (2014). Impaired Myocardium Energetics Associated with the Risk for New-Onset Atrial Fibrillation after Isolated Coronary Artery Bypass Graft Surgery. Coron. Artery Dis. 25 (3), 224–229. 10.1097/mca.0000000000000081 24463787

[B21] Wang Z. Chen Z. Wang X. Zhang L. Li S. Tian Y. (2018). The Disease Burden of Atrial Fibrillation in China from a National Cross-Sectional Survey. Am. J. Cardiol. 122 (5), 793–798. 10.1016/j.amjcard.2018.05.015 30049467

[B22] Wiersma M. Meijering R. A. M. Qi X. Y. Zhang D. Liu T. Hoogstra-Berends F. (2017). Endoplasmic Reticulum Stress Is Associated with Autophagy and Cardiomyocyte Remodeling in Experimental and Human Atrial Fibrillation. J. Am. Heart Assoc. 6 (10), e006458. 10.1161/JAHA.117.006458 29066441 PMC5721854

[B23] Wu N. Xu B. Xiang Y. Wu L. Zhang Y. Ma X. (2013). Association of Inflammatory Factors with Occurrence and Recurrence of Atrial Fibrillation: A Meta-Analysis. Int. J. Cardiol. 169 (1), 62–72. 10.1016/j.ijcard.2013.08.078 24095158

[B24] Yamac A. H. Kucukbuzcu S. Ozansoy M. Gok O. Oz K. Erturk M. (2016). Altered Expression of Micro-RNA 199a and Increased Levels of Cardiac SIRT1 Protein Are Associated with the Occurrence of Atrial Fibrillation after Coronary Artery Bypass Graft Surgery. Cardiovasc. Pathol. 25 (3), 232–236. 10.1016/j.carpath.2016.02.002 26952538

[B25] Yeh Y.-H. Kuo C.-T. Lee Y.-S. Lin Y.-M. Nattel S. Tsai F.-C. (2013). Region-Specific Gene Expression Profiles in the Left Atria of Patients with Valvular Atrial Fibrillation. Heart Rhythm 10 (3), 383–391. 10.1016/j.hrthm.2012.11.013 23183193

[B26] Yoon M. Yang P.-S. Jang E. Yu H. T. Kim T.-H. Uhm J.-S. (2019). Improved Population-Based Clinical Outcomes of Patients with Atrial Fibrillation by Compliance with the Simple ABC (Atrial Fibrillation Better Care) Pathway for Integrated Care Management: A Nationwide Cohort Study. Thromb. Haemost. 119 (10), 1695–1703. 10.1055/s-0039-1693516 31266082

[B27] Zhang P. Wang W. Wang X. Wang X. Song Y. Zhang J. (2013). Focal Adhesion Kinase Mediates Atrial Fibrosis via the AKT/S6K Signaling Pathway in Chronic Atrial Fibrillation Patients with Rheumatic Mitral Valve Disease. Int. J. Cardiol. 168 (4), 3200–3207. 10.1016/j.ijcard.2013.04.113 23639457

[B28] Zhu Y. Kuang L. Wu Y. Deng H. She H. Zhou Y. (2021). Protective Effects of Inhibition of Mitochondrial Fission on Organ Function after Sepsis. Front. Pharmacol. 12, 712489. 10.3389/fphar.2021.712489 34566637 PMC8457550

